# Application of an imaging flow cytometry γ-H2AX assay for biodosimetry using supervised machine learning

**DOI:** 10.1080/09553002.2025.2536108

**Published:** 2025-08-11

**Authors:** Eman M. Hassan, Benjamin Puzantian, Jessica M. Mayenburg, Melody Li, Mehreen Rashid, Ruth C. Wilkins, Lindsay A. Beaton-Green

**Affiliations:** a Consumer and Clinical Radiation Protection Bureau, Environmental and Radiation Health Sciences Directorate, Health Canada, Ottawa, ON, Canada; b Department of Physics, Carleton University, Ottawa, ON, Canada

**Keywords:** Imaging flow cytometry, γ-H2AX, supervised machine learning, ionizing radiation, biodosimetry, DNA damage

## Abstract

**Purpose:**

Phosphorylation of the histone H2AX (γ-H2AX) is a rapid response to radiation-induced DNA double strand breaks (DSBs) and is a good biomarker for exposure to ionizing radiation. The signal has traditionally been detected by microscopy (spot counting) or by flow cytometry (fluorescent intensity). An imaging flow cytometry (IFC) method has been developed, which combines the high resolution of microscopy with the statistical power of flow cytometry methods to measure γ-H2AX in human lymphocytes.

**Materials and Methods:**

The assay was optimized and validated for both sample acquisition and data analysis, in the dose range of 0–10 Gy. For data analysis, mean fluorescence intensity (MFI), spot count (foci per cell), and average area of the spots were used with the supervised machine learning (SML) K-Nearest Neighbors (K-NN) algorithm to estimate doses. These dose estimates were compared to the traditional flow cytometry method of estimating doses from an MFI-based dose response curve.

**Results:**

A statistical analysis of both methodologies showed that SML K-NN method was able to determine the dose delivered to blind, irradiated samples more accurately than when using a linear fit of the MFI response alone, especially in the 7–10 Gy dose range.

**Conclusions:**

The efficiency of the γ-H2AX-IFC assay, 1 hour post-exposure, has been improved and validated using the SML K-NN methodology for dose estimation. This study could help establish the γ-H2AX assay as a triage tool for the rapid screening of a large number of samples.

## Introduction

Biodosimetry is a method for estimating the amount of ionizing radiation (IR) to which an individual has been exposed by quantifying damage in biological material. Traditionally, biodosimetry assays have been based on the enumeration of cytogenetic damage in lymphocytes using methods such as the standard dicentric chromosome assay (DCA), the cytokinesis blocked micronucleus (CBMN) assay and the translocation analysis using Fluorescence *in Situ* Hybridization (FISH) (IAEA 2011). Despite their specificity, high sensitivity, and ability to perform dose estimations long times after an exposure (months to years, depending on the method), these assays are not well suited for high-throughput biodosimetry due to the time required to culture lymphocytes and prepare slides, as well as the need for skilled cytogeneticists (Lloyd et al. 2000; Swartz et al. 2014; Einbeck et al. 2018; Raavi et al. 2021; Escalona et al. 2022).

Recently, there has been significant interest in adapting these assays to imaging flow cytometry (IFC); particularly for high-throughput biodosimetry (Rodrigues et al. 2014a, 2014b; Beaton-Green and Wilkins 2016; Rodrigues et al. 2016; Beaton-Green et al. 2017; Wilkins et al. 2017; Wang et al. 2019; Beaton-Green et al. 2023). This interest has been driven by the ability of IFC to rapidly analyze damage in large cellular populations without the time-consuming need to prepare slides. IFC also provides additional imaging capabilities, such as multiple channels, imaging features and masks, that can supplement existing assays. Such capabilities offer the potential for a rapid and high-throughput assay, that can handle a large number of samples while delivering accurate dose estimates for triage dose assessment (Sullivan et al. 2013; Chaurasia et al. 2021; Raavi et al. 2021).

IR-induced DNA damage to human cells results in a variety of alterations, with double strand breaks (DSBs) being the most harmful (Chatterjee and Walker 2017). Following IR exposure to the cells, DSBs initiate DNA damage response by the rapid phosphorylation of the modified histone protein H2AX (termed γ-H2AX when phosphorylated) in regions surrounding the break (Rogakou et al. 1998; Celeste et al. 2003; Valdiglesias et al. 2013). These regions are called foci (focal sites in cell nuclei). Due to its strong correlation with DSBs and IR dose received, the γ-H2AX assay is an attractive alternative to traditional cytogenetic-based assays as the intensity of the γ-H2AX signal follows a linear response in the dose range of interest (0–10 Gy) (Andrievski and Wilkins 2009). A drawback is that the γ-H2AX signal peaks shortly after exposure (between 30 minutes to an hour) and then decreases, returning to near baseline levels after 24 hours as a result of dephosphorylation of γ-H2AX (Wang et al. 2014; Sharma et al. 2015; Einbeck et al. 2018). While this poses a major limitation of γ-H2AX as a biodosimeter, studies based on foci counting have reported the presence of residual foci above baseline in *ex vivo* irradiated lymphocytes 48 h and 96 h after IR exposure due to the failure of efficient DNA DSB repair (Redon et al. 2009; Horn et al. 2011). Furthermore, foci in in vivo irradiated non-human primates have been shown to persist for several days after exposure (Redon et al. 2010). Thus, even with the rapid kinetic decay of the signal, the γ-H2AX assay could provide rapid, same day, indication of IR exposure in triage situations (Andrievski and Wilkins 2009; Viau et al. 2015; Raavi et al. 2021). Through these studies, the γ-H2AX assay has been shown to be reliable, reproducible and amenable to adaptation to a high-throughput, automated process (Redon et al. 2009; 2011; Wilkins et al. 2017).

Many methods have been established to measure γ-H2AX in vitro, such as, immunofluorescence labeling and foci counting of microscopy images (Rothkamm, Beinke et al. 2013; Noubissi et al. 2021; Wanotayan et al. 2022). These microscope-based methods are tedious and require manual efforts to count γ-H2AX foci. Traditional flow cytometry has been demonstrated as a practical alternative to microscope scoring where the overall intensity of the γ-H2AX signal can be measured (Andrievski and Wilkins 2009; Horn et al. 2011; Wang et al. 2014; Raavi et al. 2021; Wanotayan et al. 2022) and, without the need for manual scoring, can increase sample throughput to hundreds of samples per day. IFC is a powerful tool that combines the statistical power of traditional flow cytometry with the high resolution and specificity of microscopy. In IFC, high resolution images are generated for each fluorescently-labelled cell (Rodrigues et al. 2016; Wilkins et al. 2017). These images allow for visual confirmation of γ-H2AX foci and, with an image analysis software, can be used to verify the damage resulting from IR on human cells.

Developing a γ-H2AX assay in human lymphocytes using flow cytometry has been previously reported (Roch-Lefèvre et al. 2010; Rothkamm, Barnard, et al. 2013; Rothkamm, Beinke, et al. 2013; Rothkamm, Horn, et al. 2013; Moquet et al. 2014; Durdik et al. 2021; Bacon et al. 2023). Two additional studies established γ-H2AX dose response curves based on irradiated samples between the ranges of 0–50 cGy and 0–4 Gy respectively using the IFC (Durdik et al. 2015; Lee et al. 2019). Moreover, in Lee et al. (Lee et al. 2019), apoptotic cells were excluded from γ-H2AX analysis based on fluorescence intensity and morphology. In this study, γ-H2AX foci were detected by IFC after exposure to IR for doses between 0 Gy to 10 Gy, 1 hour post-exposure. Additionally, a strategy was developed to separate healthy from non-healthy lymphocytes using a supervised machine learning (SML) module for image analysis. With the healthy lymphocytes separated, multiple γ-H2AX dose response endpoints were used with SML to estimate doses. This was done without a dose response curve by non-parametric regression (NPR). The approach incorporated the mean fluorescence intensity (MFI), average spot count (foci), and average area of the spots to accurately estimate doses with the K-nearest neighbors (K-NN) SML NPR model. The K-NN was validated by generating dose estimates by irradiating blinded samples and statistically comparing these estimates to ones from a traditional MFI-based dose response curve. Through this comparison, the K-NN model was found to perform more accurately in the higher dose regions while delivering precise dose estimates throughout the 0 Gy to 10 Gy dose region. In this paper, an IFC-based γ-H2AX assay (γ-H2AX-IFC) with the potential for high-throughput biodosimetry is described and a novel model to conduct biodosimetric dose estimations, done in the absence of dose response curves, is presented and verified with a set of blinded dose responses.

## Materials and methods

### Blood collection, sample preparation and labeling of γ-H2AX

To generate γ-H2AX dose responses, whole blood was collected from 10 healthy female and male donors (5 from each) between the ages of 20 to 60 years, with informed consent and approval by Health Canada’s Research Ethics Board (protocol REB 2002–0012H). All donors were nonsmokers, had no declared illnesses, and no known exposure to medical ionizing radiation within the last 12 months.

The assay was conducted with the following protocol: blood was drawn by venipuncture in lithium-heparinized Vacutainer^®^ tubes (BD Vacutainer^™^, Mississauga, ON, Canada). From the blood drawn from each donor, 9 × 450 μL samples were prepared and irradiated as follows: blood was first mixed with complete RPMI media (84% media (Gibco, Waltham, MA), 15% heat-inactivated FBS (Sigma-Aldrich, St. Louis, MO), and 1% L-Glutamine/Pen Strep (Sigma-Aldrich)), at 3:7 ratio of blood to complete media. Samples were transferred to 15 mL tubes (total volume of 1.5 mL for each) and transported on ice to an XRAD 320 X-ray cabinet biological irradiator (Precision X-Ray, N. Branford, CT).

Prior to irradiation, the X-ray cabinet machine was calibrated using a PTW TW30013 farmer-type ion chamber and a PTW UNIDOS Romeo electrometer (PTW Dosimetry, Lörracher Strasse 7, 79115 Freiburg; calibration co-efficient N_k_ = 48.73 mGy/nC) in the same configuration as the sample irradiation. For sample irradiation, tubes were placed on ice on their side in the middle of the cabinet on a Styrofoam plate and exposed to 250 kVp X-rays at a dose rate of 1.5 Gy/min. Two samples were used as non-irradiated controls and the remaining 7 were irradiated with 0.5, 1, 2, 4, 6, 8, and 10 Gy X-rays. The exact dose delivered was measured with the ion chamber in place during the sample exposure.

Post-irradiation, samples were incubated in a water bath at 37 °C for 1 hour and centrifuged at 500 × g, the media was removed and the pellets were resuspended. Samples were treated by adding a pre-warmed lysis/fix solution (Becton, Dickinson and Company (BD); Franklin Lakes, NJ) to each sample at a ratio of 1:20 blood to solution to lyse red blood cells and fix the lymphocytes. Samples were mixed briefly using a vortex to prevent blood clumps and were incubated for 10 min at 37 °C. Cell suspensions were centrifuged at 500 × g for 8 min, the supernatant was removed and the cell pellets resuspended in 1 mL of ice-cold 0.12% TX-100 in 1x PBS and incubated at room temperature for 30 min. Samples were washed with 1 mL 4% FBS in 1x PBS cold wash buffer and centrifuged as above. After removing the supernatant, the pellets were loosened and stored at 4 °C in the fridge overnight in preparation for antibody staining.

Samples were stained using Alexa Fluor^®^ 488 mouse anti-H2AX (pS139) (BD #560445). Briefly, this involved cell pellets being washed with 1 mL of cold TBS, centrifuged at 400 × g for 5 min at 0 °C, aspirated and washed again with cold TST (0.01% TX-100, 4% FBS in cold TBS), incubated for 10 minutes on ice and centrifuged at 400 × g for 5 min at 0 °C. The supernatant was aspirated and the pellets were resuspended by vortexing. Samples were rinsed with 1 mL cold TBS, transferred to 1.5 mL Eppendorf tubes and spun down again at 400 × g for 5 min at 0 °C. Samples were then incubated with anti-H2AX (pS139) antibody (final concentration 5 μM in 30 μL of fixed cell suspension) at room temperature for 1 hour in the dark. Subsequently, samples were washed with 1 mL of 2% FBS in TBS and centrifuged at 300 × g for 10 min at room temperature and aspirated to 40 μL for acquisition by IFC.

For the model validation tests, whole blood was collected from 11 healthy donors (6 female, 5 male), between the ages of 20 to 60 years, with informed consent. The samples were prepared and irradiated by the same protocol described above. Samples were then irradiated with X-rays at doses between 0 and 10 Gy, with the doses blinded prior to analysis and determination of the doses.

### Sample acquisition with imaging flow cytometry

**Figure 1.**
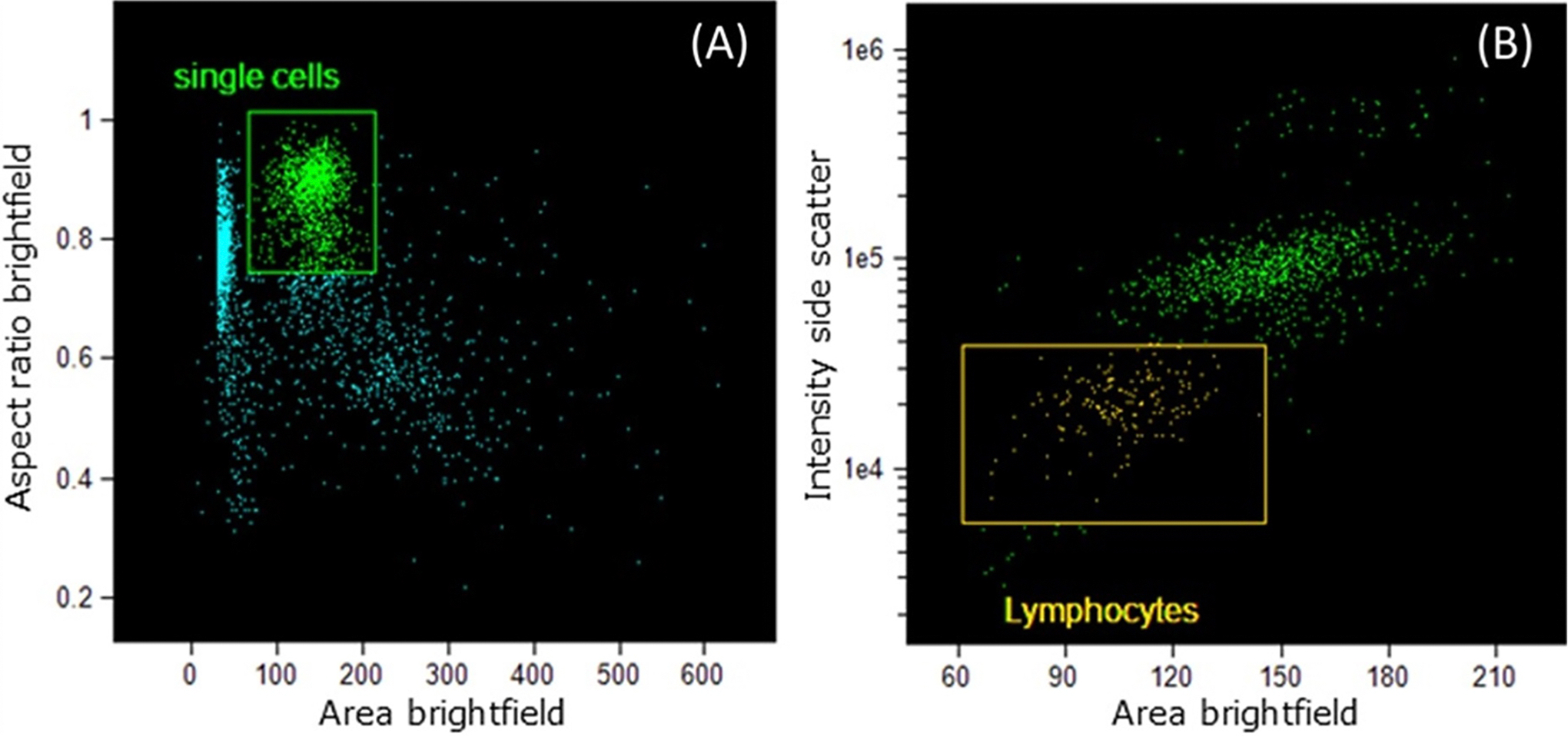
Acquisition template used to collect blood cells. Single cells selected based on the area and aspect ratio of the brightfield signal (a), and lymphocytes acquired using the area function from this single cell population based on the area of the brightfield and the intensity of the side scatter signals (B).

Sample acquisition was performed using the IFC (ImageStream^®^X Mark II (ISXMkII), Cytek Biosciences, Seattle, WA) by manually loading samples in the ISXMkII in 1.5 mL Eppendorf tubes. An acquisition template was established using ISX INSPIRE^®^ software for acquiring lymphocytes. Brightfield was set at Gradient_RMS > 55 (in-focus population) to gate out objects that were out of focus. Single cells were collected by gating based on brightfield area and aspect ratio, excluding any unwanted events like debris and doublet or triplet cells (Figure 1A). Lastly, the lymphocyte population was selected based on brightfield and side scatter features (Figure 1B).

All samples were run at 60× magnification with the 488-nm laser at 150 mW and 785-nm side scatter laser at 0.5 mW. Channel 4 was used to collect brightfield images, channel 2 for AlexaFluor^®^488 antibody signal and channel 6 for side scatter. Approximately 3,000 lymphocytes were acquired at each exposure and for control samples.

### Image analysis

Images collected with the acquisition template were subsequently analyzed using IDEAS 6.3 software. This involved developing a template to analyze several features of the lymphocyte population such as the MFI of the γ-H2AX signal in the cells, the average γ-H2AX spot count, and the average area of the spots, with the assistance of a machine learning (ML) module in the IDEAS software.

**Figure 2.**
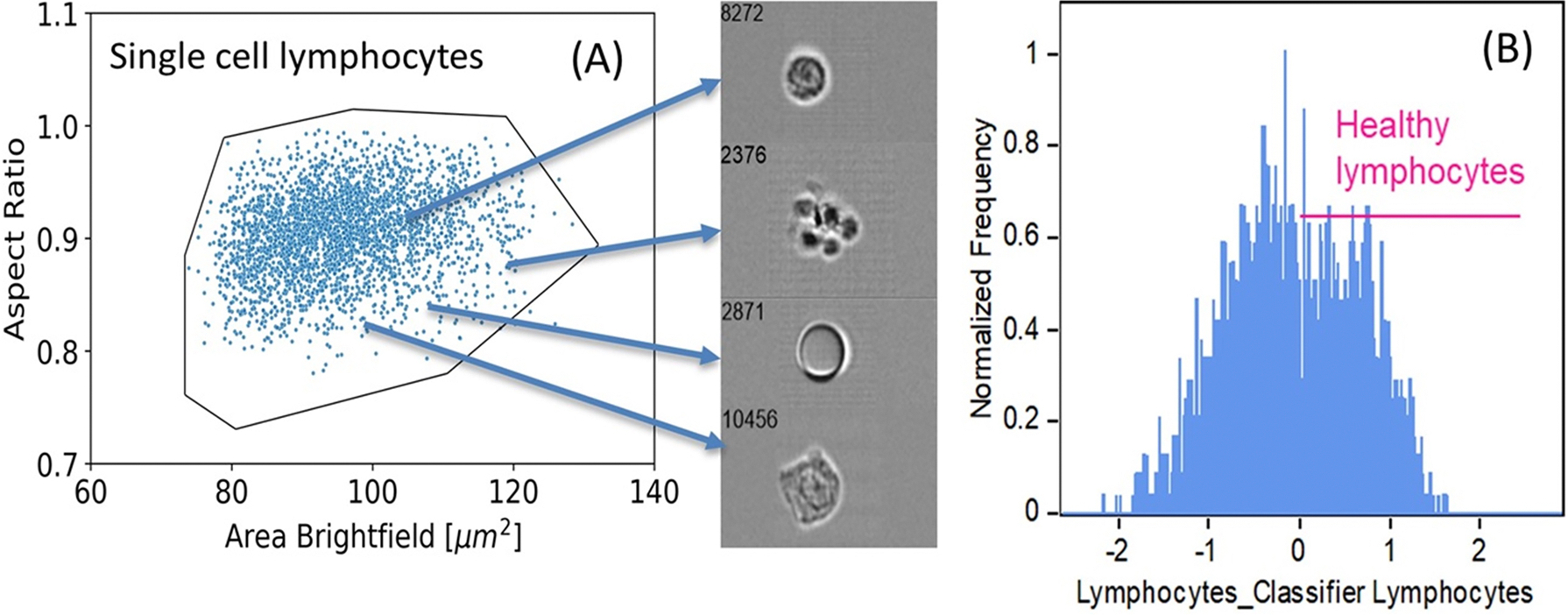
Development of machine learning function to classify healthy lymphocytes based on the IDEAS machine learning module. (A) Sample images of objects found in the lymphocyte population, where only the top object was considered a healthy lymphocyte. (B) resulting classifier for healthy lymphocytes generated by machine learning.

The lymphocyte population acquired by IFC contained several different objects of similar size and aspect ratio that were clearly not lymphocytes (Figure 2A). Healthy lymphocytes were manually classified by tagging two populations based on the brightfield images, as 1) healthy lymphocytes or 2) other particles (for example, apoptotic cells and debris). This classification was based on circularity and internal structure. These tagged populations were then trained and tested with the machine learning (ML) module in the IDEAS software, resulting in a classifier that could discriminate between the two populations and allowed a gate to be set for the healthy lymphocyte population (Figure 2B). This classifier and gate were then applied to each of the samples to restrict the analysis to only the healthy lymphocyte population.

**Figure 3.**
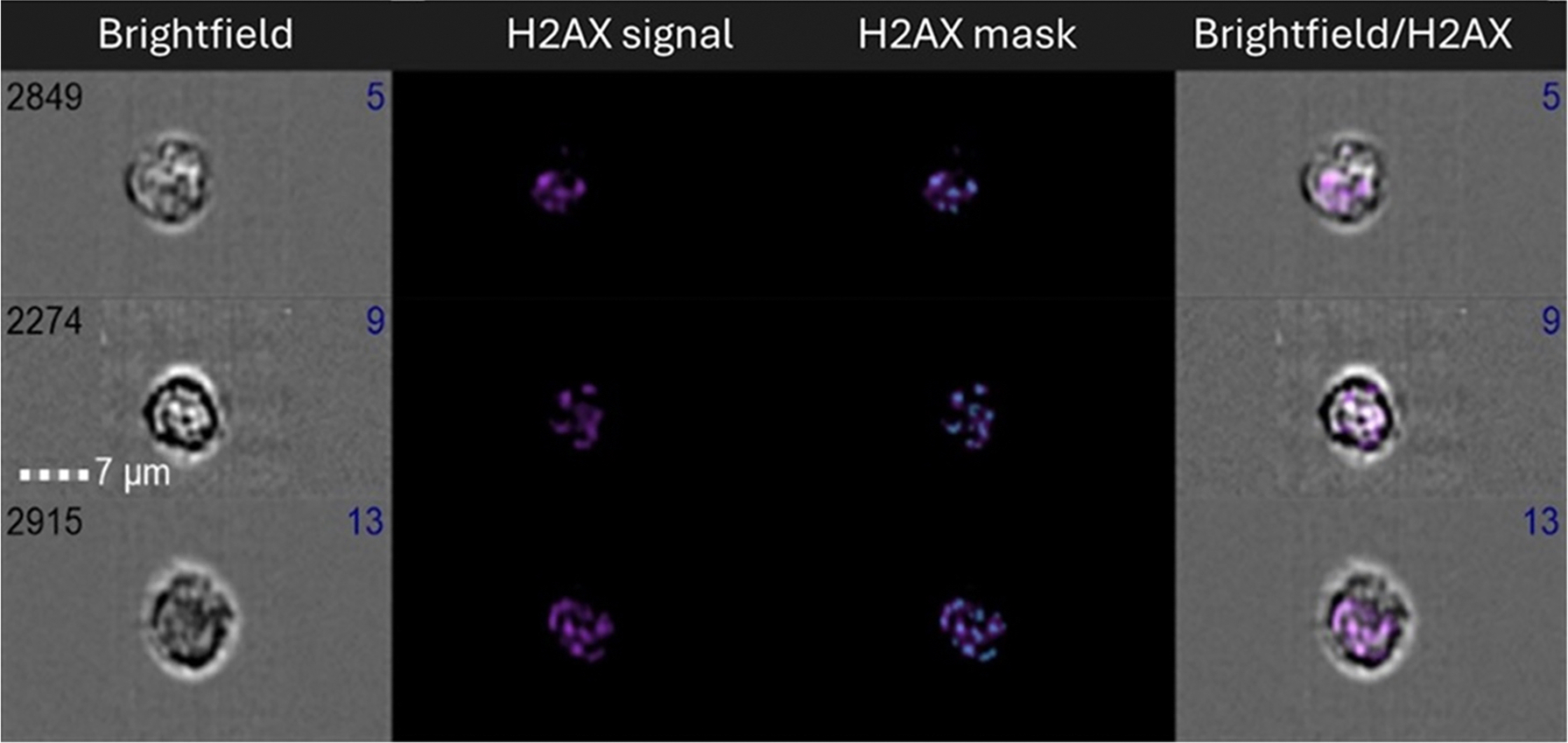
Sample of cells with the spot counting mask for γ-H2AX foci shown. Column one is the brightfield image, column 2 is the γ-H2AX signal, column 3 has the spot mask applied and column 4 shows the overlay of the γ-H2AX signal on the brightfield image. the blue numbers indicate the number of γ-H2AX foci.

From the healthy lymphocyte population, images were analyzed for three main features: MFI, spot count, and the spot area (Table 1). For the spot count feature, a mask was defined on each image in the brightfield (BF) channel around the nucleus of the lymphocytes and a spot mask for the γ-H2AX signal was then applied to this area (Figure 3).

### Dose estimation methods

#### Dose response curves

Dose response curves were generated with either features related to γ-H2AX foci or the MFI of the γ-H2AX signal. MFI dose response curves were fit as a linear response to dose:

(1) MFI=αD+β,

where D is the dose in Gy and α and β are the fitting coefficients; α is the increase in γ-H2AX fluorescence per dose and β is the average background fluorescence, respectively.

Whereas, the γ-H2AX foci (spot count and area of spot) features were empirically modeled by:

(2) $$Y=v\left(1-e^{-\xi D}\right),$$

where Y is the average number of spots counted or the average area of the spots, v is the maximum fluorescence and ξ is a coefficient controlling the level of steepness of the spot feature dose response.

All the curve fits and confidence intervals were generated using the SciPy’s curve fitting (Virtanen et al. 2020) Python package, Version 1.10.0.

### Supervised machine learning

Supervised machine learning (SML) was investigated as an alternative to dose response curves, as it takes into account multiple features extracted from the image analysis. The selection of suitable SML algorithms is described in Supplementary Material. Briefly, a tabulated dataset of the MFI, average number of spots and area of spots for each X-ray dose was split into training and testing subsets (70:30) and optimal hyperparameters were found through an iterative tuning analysis. SML algorithms were then trained with the optimal hyperparameters, dose estimates were generated for the testing subset, and models were compared. The non-parametric regression, K-Nearest Neighbors (K-NN) algorithm was found to be the most suitable model to estimate the dose based on γ-H2AX features and an optimization of the root mean squared error (RMSE). Based on the iterative error analysis for k determination described in supplementary material, k=7 was found to be the optimal parameter choice.

Once developed on the entire calibration data, the doses were estimated with the K-NN algorithm through comparison of feature data belonging to unknown doses, ${\hat{X}}_{n,m}$, to nearest neighboring calibration data dose response points in $X_{n,m}$. These nearest neighbors are constrained by the neighborhood size, k, which is found geometrically by computing the Euclidean distance between dose responses. Then the unknown dose, $\hat{D}$, is estimated by the average of the fitted dose points within k:

(3) $$\hat{D}=\frac{1}{k}\sum_{i=1}^{k}D_{i}\left(X_{n,m}\right),$$

where $D_{i}$ is the i-th dose corresponding to closest calibration data points, $X_{n,m}$.

### Statistical evaluation

For assessing the SML and dose response curve models’ precision in dose estimations, the performance was quantified in terms of residuals, ${\hat{D}}_{i}-D_{i}$, to the validation dataset doses, $D_{i}$, measured with the ionization chamber. The statistical performance metric used for this purpose was the root mean squared error (RMSE):

(4) $$RMSE=\sqrt{\frac{1}{n}\sum_{i=1}^{n}\;{\left({\hat{D}}_{i}-D_{i}\right)}^{2}},$$

A blinded validation was conducted where dose estimates were assessed in terms of being within the ranges of ±0.5 Gy and ±1 Gy of the X-ray dose delivered to the samples. This range constitutes acceptable dose ranges for low (<3 Gy) and high (>3 Gy) doses respectively (Lloyd et al. 2000).

## Results

To validate this *γ-*H2AX-IFC assay, the first step was to generate dose responses for different endpoints. Then dose estimations on the blinded samples were made using the MFI dose response curve alone and by combing dose responses of multiple endpoints with the K-NN model. These results were statistically compared to determine optimal model choice.

### γ-H2AX dose response curves

**Figure 4.**
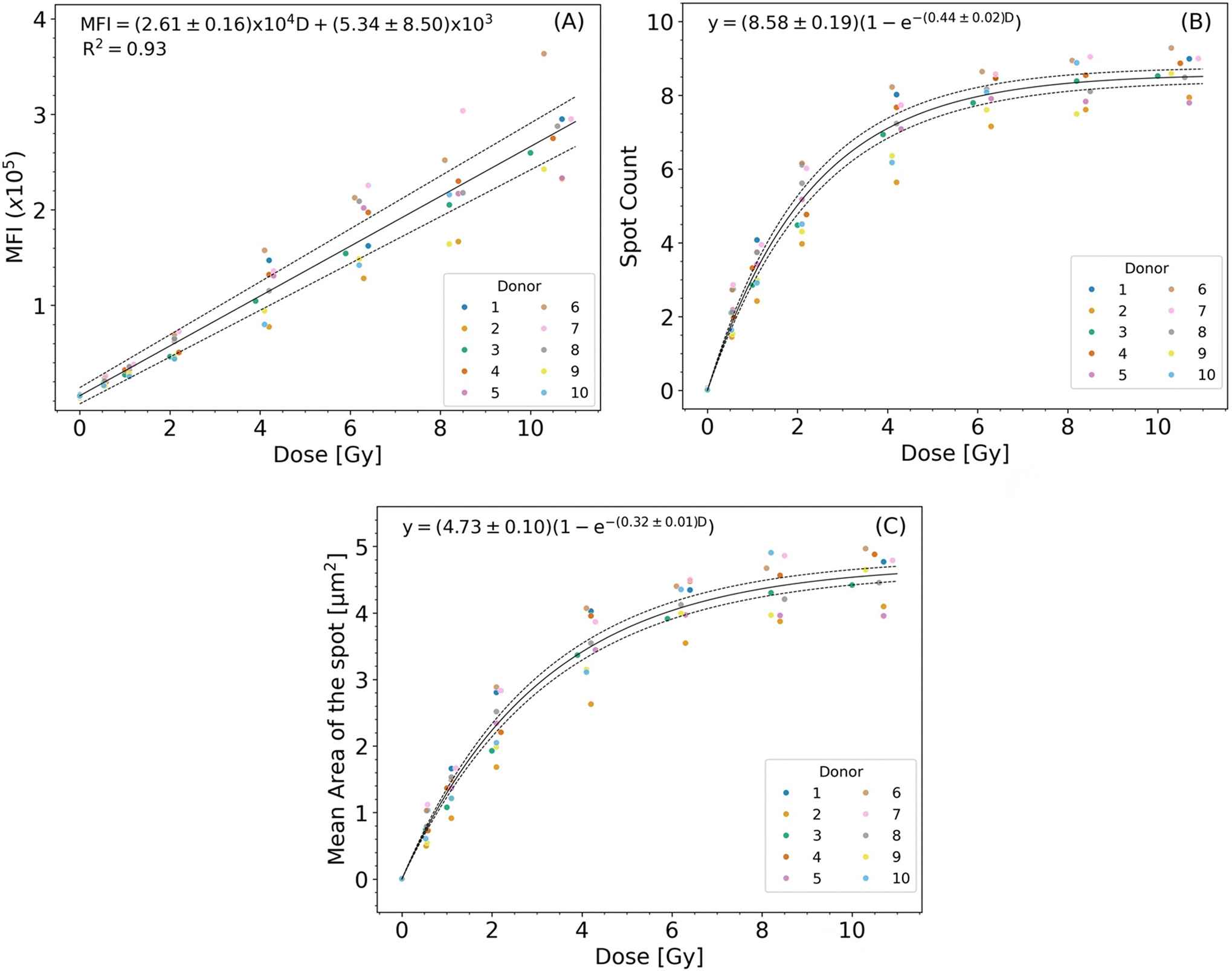
Dose responses of (A) MFI, (B) number of spot counts and (C) mean area of the spots of γ-H2AX in human lymphocytes 1 hour post irradiation. All plots consist of 10 donors. Solid black lines represent curve fits. The dotted lines represent the 95% confidence intervals (CIs).

Figure 4 shows the dose response curves based on the MFI, spot counts, and area of the spots. There is a linear relationship between MFI and dose (Figure 4A), and an exponential plateau relationship with both the spot count (Figure 4B) and average area of the spots (Figure 4C). Based on these results, the MFI was chosen as the best single dose response feature for calibration curve (CC) dose estimation.

### Dose estimations

**Figure 5.**
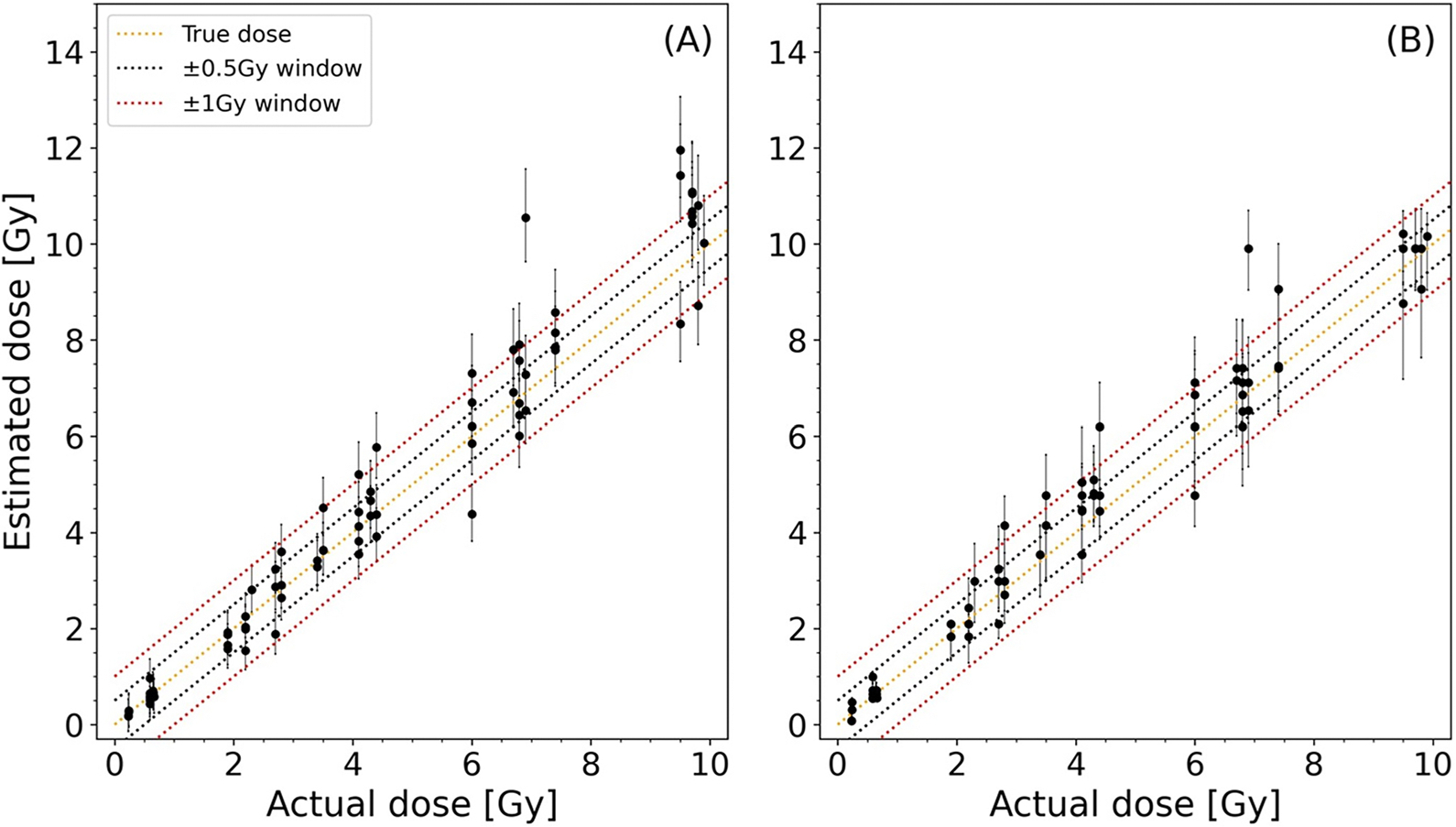
Validation plots of dose estimates based on (A) MFI dose response curve and (B) K-NN SML with *k = 7* for γ-H2AX in human lymphocytes 1 hour post exposure. Error bars in (A) and (B) depict 95% CIs. In (B), the 95% CIs are found by bootstrapping as described in the supplemental online material.

**Figure 6.**
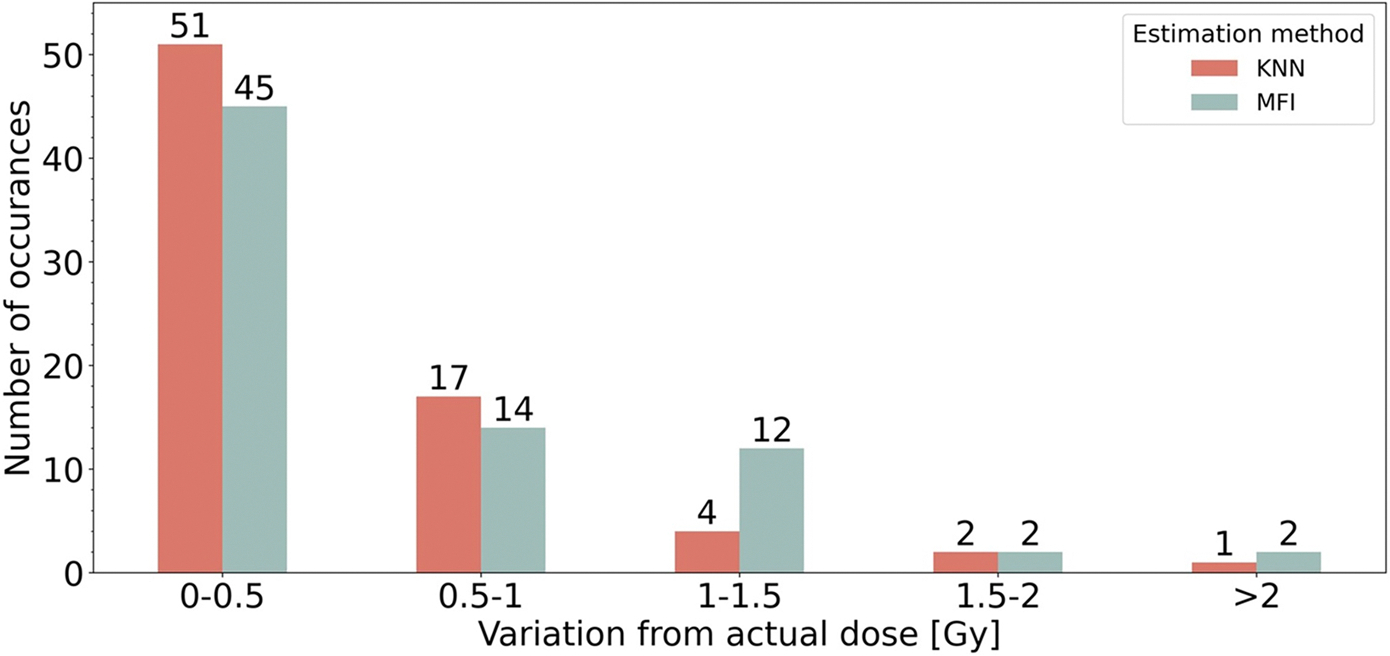
The bars indicate the number of dose estimates falling within the indicated ranges of the X-ray dose delivered to the samples of validation data for each dose estimation method. The exact number of instances is written above the bar.

**Figure 7.**
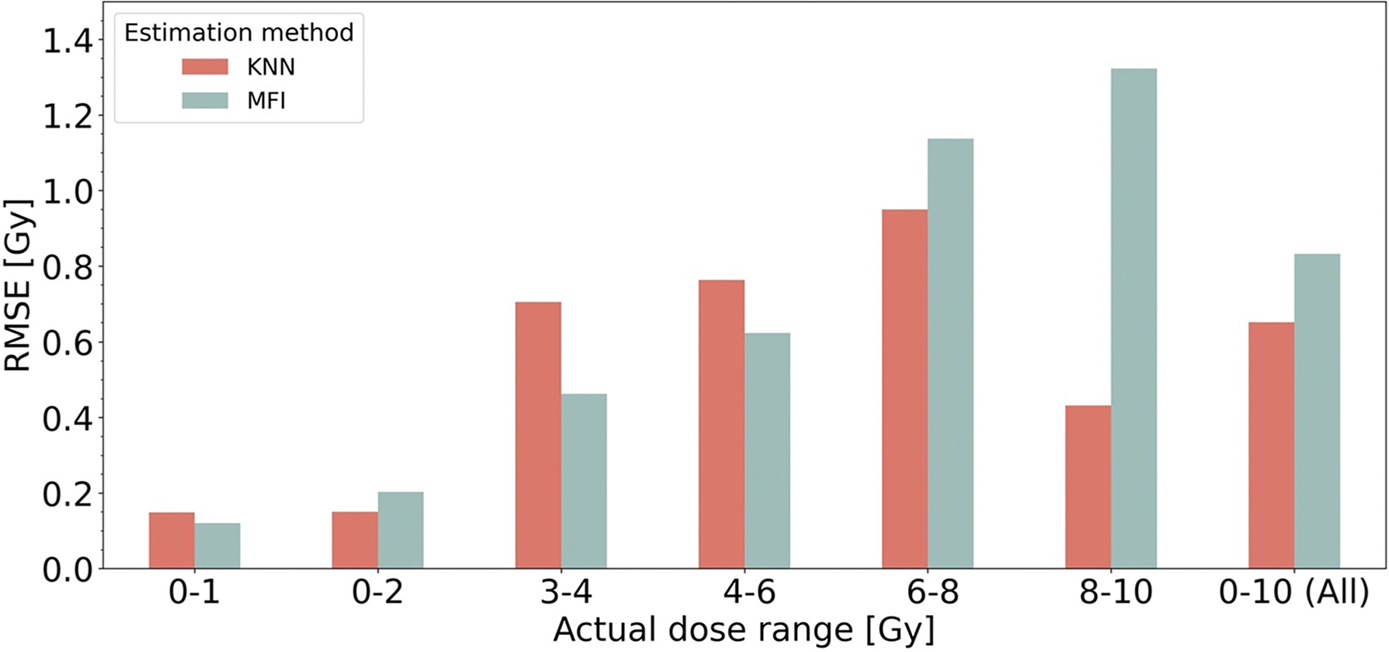
The bars represent the RMSE of doses as determined by each estimation method over different dose ranges.

In Figure 5, dose estimates from 75 blinded validation samples from nine healthy donors (separate to the population) are plotted against the X-ray dose delivered to the samples using the linear fit to the MFI dose response CC and K-NN SML approaches. Moreover, in Figure 6 and 7, the error metrics described in section 2.4. are used to evaluate the dose estimates in Figure 5. Results showed 68% and 91% of dose estimates falling within ±0.5 Gy and ±1 Gy respectively of the X-ray dose delivered to the samples when the K-NN SML methodology was used, while the dose estimates by the MFI CC resulted in 60% and 79%, respectively, falling within the same ranges (Figure 6). The RMSE increased with dose reaching a peak of 1.3 Gy RMSE for the MFI approach, whereas the RMSE for the K-NN approach had a smaller overall increase for the low doses, peaking at about 1.0 Gy and decreased in the 8–10 Gy region, implying improved response in the 6–10 Gy region. While the MFI method performed similarly to or better than the K-NN method for doses under 6 Gy, overall, the RMSE error obtained from the K-NN method was lower than from MFI dose response curve methodology, indicating better dose estimations (Figure 7).

### Dose classifications

**Figure 8.**
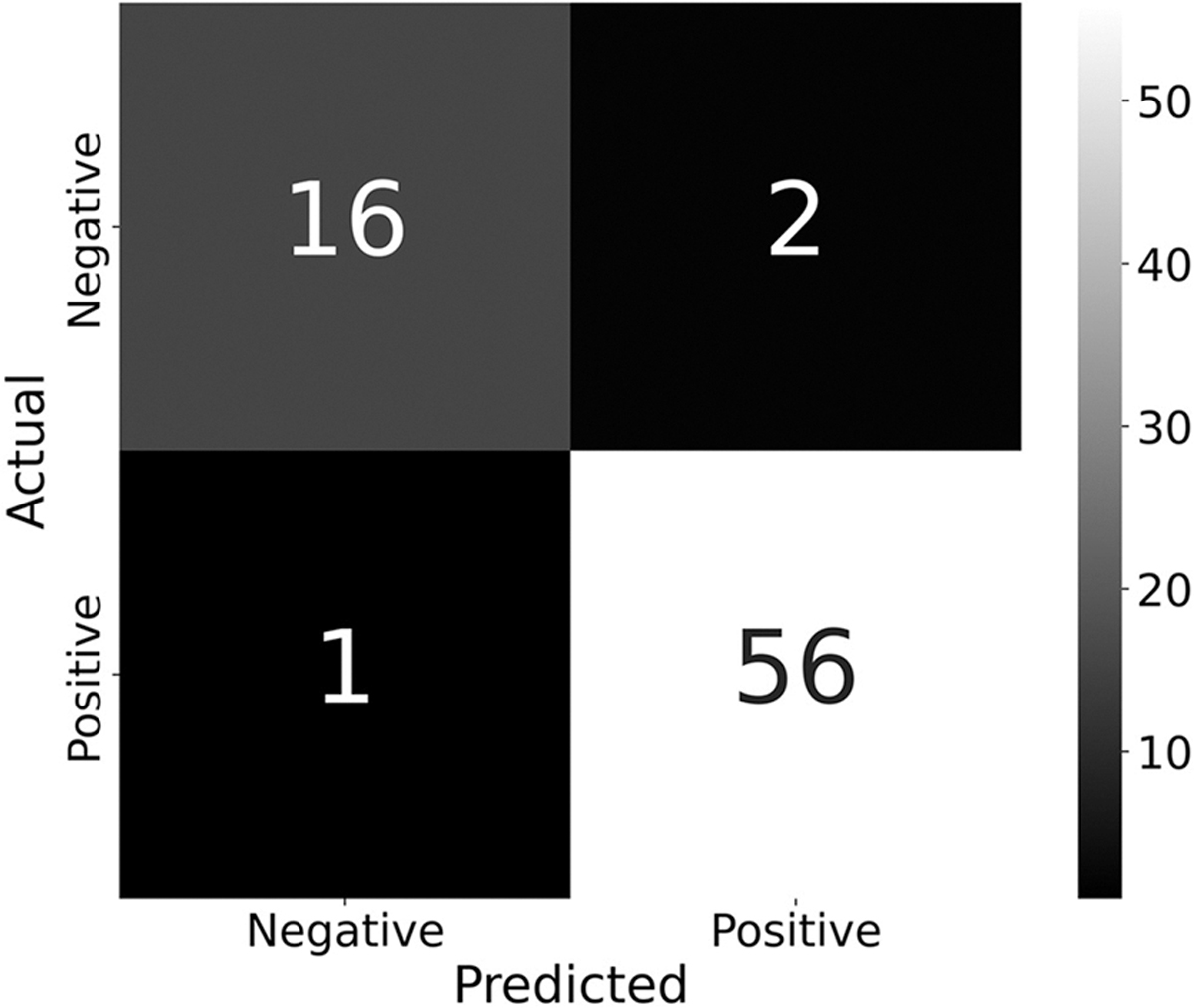
A confusion matrix denoting number of true and false positive and negatives. The first row of the matrix represents the number of true negative and false positive classifications, respectively, while the second row represents the number of false negative and true positive classifications.

Figure 8 presents dose estimations generated by the SML K-NN model, classified as positive or negative based on a threshold for positive set at 2 Gy or greater. Based on the 75 samples conducted during the validation experiments (Figure 5), the classifier exhibits a high level of precision (94% for negative and 97% for positive) and recall (89% for negative and 97% for positive) as shown in Figure 8 resulting in a low number of false positives (1) and false negatives (2). Overall, the classifier does remarkably well in classifying doses based on a 2 Gy threshold, evident by the very high accuracy of 96%.

## Discussion

The phosphorylation of the histone H2AX has been demonstrated to be a robust indicator of DNA double strand breaks and, therefore, has potential as a biodosimetry method for estimating doses to individuals who have been possibly exposed to ionizing radiation (Roch-Lefèvre et al. 2010; Moquet et al. 2014). This has been previously explored by several groups (Rothkamm, Barnard, et al. 2013; Rothkamm, Beinke, et al. 2013) and tested in some inter-laboratory comparisons (ILCs) (Rothkamm, Horn et al. 2013).

This work has focused on optimizing a γ-H2AX method for dose estimations developed for IFC. This was accomplished by combining data analysis from IFC and the K-NN SML algorithm. Building on previously reported γ-H2AX-IFC assays (Durdik et al. 2015; Lee et al. 2019; Durdik et al. 2021), the protocol was optimized and a dose estimation tool was created and validated for dose estimations up to 10 Gy.

The first improvement to the protocol toward maximizing the efficiency of data collection and analysis was to optimize the acquisition by collecting only lymphocytes as compared to the standard method of collecting all single cells reported elsewhere (Geißler et al. 2019; Lee et al. 2023). The improved acquisition template allows for faster analysis time with IDEAS software by decreasing the files size to 50% compared to collecting all leucocytes. The data analysis was then improved by removing apoptotic cells and other non-lymphocytes using the machine learning module in IDEAS similar to previously reported methods (Bacon et al. 2023). This focused the analysis on healthy lymphocytes, reducing any artifacts that could be caused by analysis of apoptotic cells and other non-lymphocytes.

Previous work has been published using γ-H2AX-IFC assay based on counting γ-H2AX foci per cell as a result of DNA damage after irradiation of whole blood samples up to 4 Gy (i.e., spot counting) (Lee et al. 2019; Durdik et al. 2021). In this dose range, a linear response between spot count and dose was reported, however, this breaks down at higher doses (7–10 Gy) as more DNA damage results in more closely spaced spots that form clusters inside the nuclei of damaged cells. In our study, and for the first time, we developed an approach that takes into consideration not only MFI but the spot count and the average area of the spots as indicators of the dose estimation. When a 2-dimensional image of a 3-dimensional cell is captured, some spots may be co-located and counted as a single spot. This effect was clearly seen in Figure 4B and C, where at high doses when both the spot count and the area of the spots was measured as a function of dose. A fairly linear response was observed at lower doses (<4 Gy) which approached a plateau value at higher doses. This had little effect on the overall MFI of the cells as the overall intensity of the γ-H2AX signal was still detected as seen by fairly linear dose response of the MFI over the entire 10 Gy dose range averaged over all donors.

Our new approach, utilized all three outputs (MFI, spot count, and average area of the spots) from IFC analysis in an SML model to incorporate more data and, therefore, yield a better dose estimation than using a single endpoint.

By comparing the dose estimates generated using the fit of the dose response curve based on MFI and those generated using the K-NN fit, it was found that the K-NN model was the best model as it was more accurate with more sample estimates falling within ±0.5 and ±1.0 Gy (Figure 6). Furthermore, the RMSE was generally improved for the K-NN SML as compared to the MFI approach, particularly at high doses. It should be noted that the MFI approach performed well in the lower dose regions, indicating that the MFI approach is valid and precise within 0–6 Gy but loses accuracy for dose assessments higher than 6 Gy (Figure 7). This is a direct result of the differences in statistical modeling between the methodologies: the MFI dose response curve has larger inter-individual fluctuations in MFI data in the higher dose region (7–10 Gy). As a result, the residuals have non-constant variance and hence the observations will unevenly weigh observations creating a less accurate line fit in higher dose regions. With the K-NN methodology, dose estimates are inferred from the data using multiple endpoints and without reference to a regression line. While not resolving the spot clustering image recognition issue, it did provide more precise dose estimates. Therefore, by including the MFI, spot count, and mean area of the spot of γ-H2AX dose responses after non-healthy lymphocyte cells were removed, with the K-NN SML methodology, a predictive model based on γ-H2AX dose responses was successfully built for doses up to 10 Gy.

As γ-H2AX is an excellent rapid biomarker of radiation-induced DNA damage, it could be useful in classifying exposure of subjects to radiation based on their dose for the rapid screening of large populations in emergency radiation responses scenarios (Escalona et al. 2022). The dose estimations from the K-NN SML model were classified based on a 2 Gy threshold, which is derived from a common tiered based threshold for radiation emergencies (Escalona et al. 2022). Under such criteria, if an individual were exposed to a radiation dose less then 2 Gy they would be classified as negative for further cytogenetic analysis and triaged into a tier I group, allowing them to return home. On the other hand, if the individual were to be exposed to a radiation dose greater than 2 Gy then they would be placed into the tier II group; resulting in further cytogenetic testing and medical intervention. This classifier performed well in triaging doses at this threshold, which was apparent from a 96% model accuracy and a low number of false positives (1) and negatives (2) in the set of 75 samples from the validation experiments. In addition to this high classification accuracy, the K-NN model’s ability to estimate doses with reasonable precision up to 10 Gy may allow for the allocation of appropriate care to persons exposed to very high doses, whom may need immediate care. The inclusion of additional features and timepoints could help to improve the classifier and extend its applicability beyond the 1-hour timepoint.

It is recognized that one major drawback of using this assay as a biodosimeter is the rapidly changing kinetics of the γ-H2AX. This means that one needs to receive a blood sample from the exposed individual within hours of the exposure and even then, an accurate dose estimate would be difficult, unless the sample was received exactly one hour after exposure. To improve upon this situation, a laboratory should have several dose response models for different times post-exposure so that the most appropriate models could be used for the dose estimate. Even with many dose models, accurate dose estimates would be difficult. For this reason, the application of this assay would act as an indicator of exposure as opposed to an accurate dosimeter. However, with the rapid throughput method described here, exposed individuals could be quickly identified and more accurate, but more labor-intensive methods could be used to provide a better estimate of the dose. Even for this use, it is important to have the assay optimized and validated in ideal conditions before its application in less optimal situations.

## Conclusions

A rapid method to estimate radiation doses based on γ-H2AX dose responses from 0 Gy to 10 Gy using imaging flow cytometry (IFC) has been developed. The assay has been validated through the analysis of blinded, irradiated samples at different doses using the K-NN SML model and MFI approaches. Both methods were able to estimate doses correctly in the dose range 0–6 Gy, however, between 7 and 10 Gy, the dose estimates based only on the MFI of the γ-H2AX signal were less accurate than those generated with K-NN SML, which incorporated multiple endpoints. Therefore, by optimizing the γ-H2AX- IFC assay, deriving multiple features based on the γ-H2AX signal, and using these features to develop the K-NN SML model, dose estimations were more accurate than by the traditional MFI calibration curve method. The γ-H2AX-IFC assay presented here could help establish a high-throughput model to triage a large number of samples in the case of large-scale radiological or nuclear incidents.

## Supplementary Material

Supp 1

Supplemental data for this article can be accessed online at https://doi.org/10.1080/09553002.2025.2536108.

## Figures and Tables

**Table 1.** Descriptions of the masks used to define the features used for the analysis. IFC channels 2 and 4 (Ch02 and Ch04) represent the γ-H2AX signal and brightfield, respectively.

Feature	Description

MFI	Intensity_MC_Ch02, Mean, gH2AX+ & Single Cells & In Focus
Spot Count	Spot Count_Erode(object(M04, Ch04, Tight), 4) and Peak(M02, Ch02, Bright, 10)_8, Mean, gH2AX+ & Single Cells & In Focus
Spot Area	Area_Erode(Object(M04, Ch04, Tight), 4) and Peak(M02, Ch02, Bright, 10), Mean, gH2AX+ & Single Cells & In Focus

## Data Availability

The data that support the findings of this study are available from the corresponding author, LABG, upon reasonable request.
