# Supplementary material for: Application of an imaging flow cytometry γ-H2AX assay for biodosimetry using supervised machine learning
Source: Int J Radiat Biol. Author manuscript; Available in PMC 2025 Sep 23. (PMC12453548; doi:10.1080/09553002.2025.2536108)
Supplement: Supp 1 [file NIHMS2105514-supplement-Supp_1.docx]

# Supplemental online material

## Supervised machine learning

The testing, training, and selection of the supervised machine learning (SML) algorithms of section 3.4.2. are expanded on here. For SML, three features were selected for multi-parametric analysis and were linked, *a priori*, to dose responses of H2AX phosphorylation. These features definitions and extraction methods are described in Section 3.3.

### Algorithm selection

The first step to SML analysis required pre-processing the raw data, which was completed by formatting the data from each experiment identically and importing the data into Python with the Pandas package, version 2.2.2. (McKinney 2010). In the dataset the donor identification number was dropped and the subsequent cleaned γ-H_2_AX dataset was separated into the matrices consisting of features $X_{n,m}$; the MFI, and the average count and area of the spots, and doses $D_{n}$, of dimensions $n x m$ and $n$, respectively. The matrix *n* represents number of features, *n = 3* while *m* is the number of observations, *m* = 80 (the 7 dose points and a control for each donor’s samples).The dataset was then split into training and testing subsets (70:30 split with a random seed of 101 chosen to reduce bias) using the Python’s Scikit-Learn package (Fabian 2011).

### Hyperparameter analysis

Algorithm-specific parameters were identified through an iterative tuning process. To ensure optimal model bias-variance, iterative hyperparameter analyses were conducted by tuning candidate algorithm’s hyperparameters as a function of RMSE on both training and testing datasets. As the K-NN model was ultimately chosen as the ideal algorithm, the selection of the optimal neighbourhood size, *k* is provided as an example.

The K-NN algorithm was iteratively tested and trained on different *k* values to find the *k* of highest stability. For both the training and testing datasets, the root-mean squared error (RMSE) was computed with the residuals of the respective doses in each of the subsets and generated dose estimations. In Figure A1, testing and training error is plotted for different *k* values; in the region *k* = 4 until *k* = 7 the training and testing RMSE are nearly equal while after *k* = 10, the error rapidly increases. Therefore, *k* = 7, having the lowest difference in errors, is the most robust choice of hyperparameter.


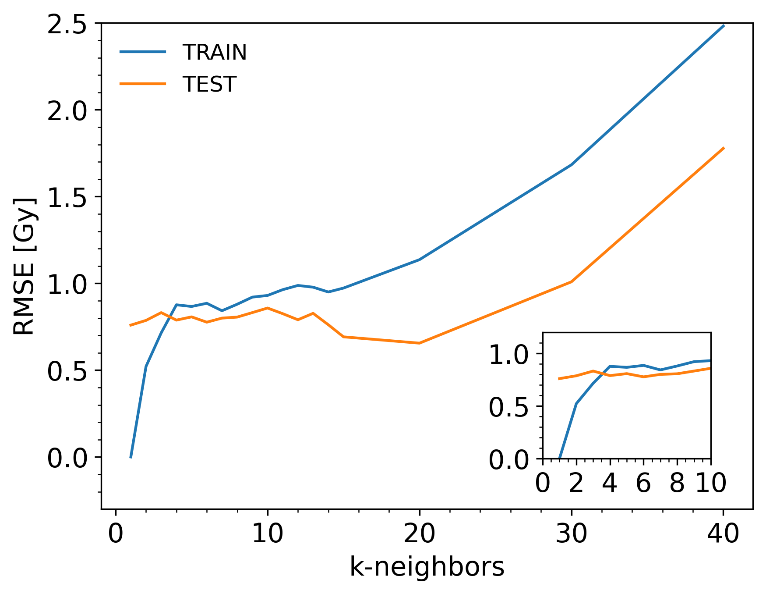


Figure A1: RMSE error of dose estimations for different *k* values. The default K-NN hyperparameters: uniform weights and the Euclidean distance metric were used. The insert shows the most stable error region, between $k=4$ until $k=7$.

### Bootstrapped 95% confidence intervals

With the K-NN algorithm selected and developed, the confidence intervals (CIs) on the dose estimates of exposed individuals were generated by the bootstrapping method.

Bootstrapping is a method that repeatably resamples from a dataset to create a large amount of data from a small dataset. With a the bootstrapping approach, the 95% confidence intervals (CIs) (Ramachandran, Kandethody M. 2020) on the dose estimates can be found for SML algorithms. First, a large amount of estimated doses are generated by randomly resampling, over many iterations, with replacement from the same set of pre-flight features and then re-fitted, with optimal hyperparameters, creating a normal distribution. Then the upper and lower 95% CIs were taken as the 2.5 and 97.5th percentiles of the normal distribution. The CIs presented in this work were generated with 10,000 bootstrap iterations.
